# *N*′-(2-Hy­droxy-4-meth­oxy­benzyl­idene)-4-methyl­benzohydrazide

**DOI:** 10.1107/S1600536812005156

**Published:** 2012-02-10

**Authors:** Yan Zhang, Min Liu, Jing-Jun Ma

**Affiliations:** a Hebei Key Laboratory of Bioinorganic Chemistry, College of Sciences, Agricultural University of Hebei, Baoding 071001, People’s Republic of China

## Abstract

The asymmetric unit of the title compound, C_16_H_16_N_2_O_3_, contains four independent mol­ecules with different conformations; the dihedral angles between the two benzene rings in the mol­ecules are 39.7 (3), 45.4 (3), 50.6 (3) and 51.6 (3)°. Intramolecular O—H⋯N hydrogen bonds are observed in the molecule. In the crystal, N—H⋯O hydrogen bonds link the mol­ecules into two crystallographically independent chains propagating in [010], and each chain is formed by two alternating independent mol­ecules. Weak C—H⋯O inter­actions also occur.

## Related literature

For the biological activities of benzohydrazide compounds, see: El-Sayed *et al.* (2011 ▶); Horiuchi *et al.* (2009 ▶). For the coordination structures of benzohydrazide compounds, see: El-Dissouky *et al.* (2010 ▶); Zhang *et al.* (2010 ▶). For normal values of bond lengths, see: Allen *et al.* (1987 ▶). For the crystal structures of similar compounds, see: Suleiman Gwaram *et al.* (2010 ▶); Liu *et al.* (2011 ▶); Zhou *et al.* (2011 ▶).
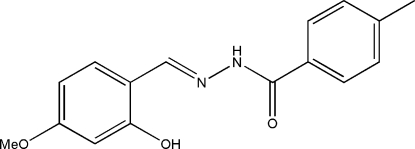


## Experimental

### 

#### Crystal data

C_16_H_16_N_2_O_3_
*M* *_r_* = 284.31
Monoclinic, 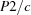

*a* = 24.871 (2) Å
*b* = 10.235 (1) Å
*c* = 23.855 (2) Å
β = 103.646 (2)°
*V* = 5901.0 (9) Å^3^
*Z* = 16
Mo *K*α radiation
μ = 0.09 mm^−1^
*T* = 298 K
0.17 × 0.13 × 0.13 mm

#### Data collection

Bruker SMART 1K CCD area-detector diffractometer
Absorption correction: multi-scan (*SADABS*; Sheldrick, 1996 ▶) *T*_min_ = 0.985, *T*_max_ = 0.988
44105 measured reflections
11954 independent reflections
5160 reflections with *I* > 2σ(*I*)
*R*_int_ = 0.095

#### Refinement

*R*[*F* ^2^ > 2σ(*F* ^2^)] = 0.083
*wR*(*F* ^2^) = 0.224
*S* = 1.02
11954 reflections
780 parameters
4 restraints
H atoms treated by a mixture of independent and constrained refinement
Δρ_max_ = 0.22 e Å^−3^
Δρ_min_ = −0.22 e Å^−3^

### 

Data collection: *SMART* (Bruker, 2007 ▶); cell refinement: *SAINT* (Bruker, 2007 ▶); data reduction: *SAINT*; program(s) used to solve structure: *SHELXS* (Sheldrick, 2008 ▶); program(s) used to refine structure: *SHELXL* (Sheldrick, 2008 ▶); molecular graphics: *SHELXTL* (Sheldrick, 2008 ▶); software used to prepare material for publication: *SHELXTL*.

## Supplementary Material

Crystal structure: contains datablock(s) I, global. DOI: 10.1107/S1600536812005156/cv5243sup1.cif

Structure factors: contains datablock(s) I. DOI: 10.1107/S1600536812005156/cv5243Isup2.hkl

Supplementary material file. DOI: 10.1107/S1600536812005156/cv5243Isup3.cml

Additional supplementary materials:  crystallographic information; 3D view; checkCIF report

## Figures and Tables

**Table 1:** Hydrogen-bond geometry (Å, °)

*D*—H⋯*A*	*D*—H	H⋯*A*	*D*⋯*A*	*D*—H⋯*A*
O2—H2⋯N2	0.82	1.93	2.649 (4)	146
O5—H5*A*⋯N6	0.82	1.88	2.606 (5)	147
O8—H8⋯N3	0.82	1.85	2.580 (4)	147
O11—H11⋯N8	0.82	1.92	2.638 (5)	146
N4—H4⋯O6^i^	0.90 (1)	2.08 (1)	2.965 (5)	171 (4)
N5—H5⋯O9^ii^	0.89 (1)	2.05 (1)	2.932 (5)	170 (4)
N1—H1⋯O12^iii^	0.90 (1)	2.10 (1)	2.984 (4)	169 (4)
N7—H7⋯O3	0.90 (1)	2.14 (1)	3.039 (5)	178 (4)
C6—H6⋯O4^iv^	0.93	2.56	3.452 (5)	160 (4)
C24—H24⋯O6^i^	0.93	2.58	3.374 (5)	143 (4)
C35—H35⋯O1^v^	0.93	2.56	3.436 (5)	157 (4)
C39—H39⋯O9^ii^	0.93	2.52	3.319 (5)	144 (4)
C19—H19⋯O10^vi^	0.93	2.52	3.429 (5)	164 (4)
C53—H53⋯O7^vi^	0.93	2.37	3.266 (5)	161 (4)

Symmetry codes: (i) 
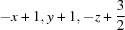
; (ii) 
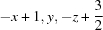
; (iii) 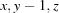; (iv) 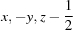; (v) 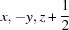; (vi) 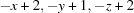.

**Fig. 1.**
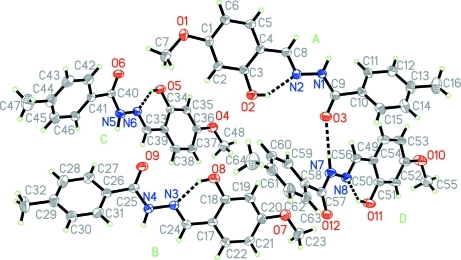
The molecular structure of the title compound, with the numbering scheme and displacement ellipsoids drawn at the 30% probability level. Hydrogen bonds are shown as dashed lines.

**Fig. 2.**
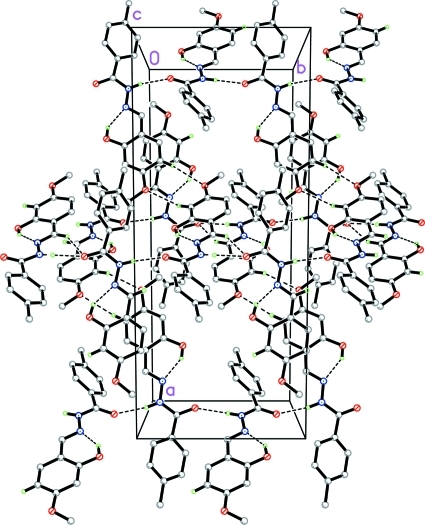
A portion of the crystal packing viewed approximately down the c axis. Dashed lines denote hydrogen bonds. H-atoms not involved in the hydrogen bonding have been omitted for clarity.

## supplementary crystallographic information

### Comment

Benzohydrazide compounds are well known for their biological activities (El-Sayed *et al.*, 2011; Horiuchi *et al.*, 2009). In addition, benzohydrazide compounds have also been used as versatile ligands in coordination chemistry (El-Dissouky *et al.*, 2010, Zhang *et al.*, 2010). As a contribution to a structural study of hydrazone compounds, we present here the crystal structure of the title compound, that was obtained as the product of the reaction of 2-hydroxy-4-methoxybenzaldehyde with 4-methylbenzohydrazide in methanol.

The asymmetric unit of the title compound contains four independent molecules with different conformations - the dihedral angles between the two benzene rings in the molecules A, B, C, and D are 39.7 (3), 45.4 (3), 50.6 (3) and 51.6 (3)°, respectively. The bond distances and angles are within normal ranges (Allen *et al.*, 1987), and agree well with the corresponding bond distances and angles reported for closely related compounds (Suleiman Gwaram *et al.*, 2010; Liu *et al.*, 2011; Zhou *et al.*, 2011).

Intermolecular N—H···O hydrogen bonds (Table 1) link the molecules into two crystallographically independent chains propagating in [010], and each chain is formed by two alternating independent molecules. Weak intermolecular C—H···O interactions (Table 1) consolidate further the crystal packing (Fig. 2).

### Experimental

To a methanol solution (20 ml) of 2-hydroxy-4-methoxybenzaldehyde (0.1 mmol, 15.6 mg) and 4-methylbenzohydrazide (0.1 mmol, 15.0 mg), a few drops of acetic acid were added. The mixture was refluxed for 1 h and then cooled to room temperature. The white crystalline solid was collected by filtration, washed with cold methanol and dried in air. Single crystals, suitable for X-ray diffraction, were obtained by slow evaporation of a methanol solution of the product in air.

### Refinement

N-bound H atoms were located in a difference Fourier map and were refined with a distance restraint, N—H = 0.90 (1) Å. The O- and C-bound H atoms were geometrically positioned (C—H = 0.93 - 0.96 Å; O—H = 0.82 Å), and refined as riding, with *U*_iso_(H) = 1.2-1.5 *U*_eq_(C, O).

### Crystal data

**Table:**

C_16_H_16_N_2_O_3_	*F*(000) = 2400
*M**_r_* = 284.31	*D*_x_ = 1.280 Mg m^−^^3^
Monoclinic, *P*2/*c*	Mo *K*α radiation, λ = 0.71073 Å
*a* = 24.871 (2) Å	Cell parameters from 2886 reflections
*b* = 10.235 (1) Å	θ = 2.5–24.5°
*c* = 23.855 (2) Å	µ = 0.09 mm^−^^1^
β = 103.646 (2)°	*T* = 298 K
*V* = 5901.0 (9) Å^3^	Block, colorless
*Z* = 16	0.17 × 0.13 × 0.13 mm

### Data collection

**Table:**

Bruker SMART 1K CCD area-detector diffractometer	11954 independent reflections
Radiation source: fine-focus sealed tube	5160 reflections with *I* > 2σ(*I*)
Graphite monochromator	*R*_int_ = 0.095
ω scan	θ_max_ = 26.4°, θ_min_ = 2.1°
Absorption correction: multi-scan (*SADABS*; Sheldrick, 1996)	*h* = −31→31
*T*_min_ = 0.985, *T*_max_ = 0.988	*k* = −12→12
44105 measured reflections	*l* = −29→28

### Refinement

**Table:**

Refinement on *F*^2^	Primary atom site location: structure-invariant direct methods
Least-squares matrix: full	Secondary atom site location: difference Fourier map
*R*[*F*^2^ > 2σ(*F*^2^)] = 0.083	Hydrogen site location: inferred from neighbouring sites
*wR*(*F*^2^) = 0.224	H atoms treated by a mixture of independent and constrained refinement
*S* = 1.02	*w* = 1/[σ^2^(*F*_o_^2^) + (0.0563*P*)^2^ + 5.6542*P*] where *P* = (*F*_o_^2^ + 2*F*_c_^2^)/3
11954 reflections	(Δ/σ)_max_ < 0.001
780 parameters	Δρ_max_ = 0.22 e Å^−^^3^
4 restraints	Δρ_min_ = −0.22 e Å^−^^3^

### Special details

**Table:**

Geometry. All e.s.d.'s (except the e.s.d. in the dihedral angle between two l.s. planes) are estimated using the full covariance matrix. The cell e.s.d.'s are taken into account individually in the estimation of e.s.d.'s in distances, angles and torsion angles; correlations between e.s.d.'s in cell parameters are only used when they are defined by crystal symmetry. An approximate (isotropic) treatment of cell e.s.d.'s is used for estimating e.s.d.'s involving l.s. planes.
Refinement. Refinement of *F*^2^ against ALL reflections. The weighted *R*-factor *wR* and goodness of fit *S* are based on *F*^2^, conventional *R*-factors *R* are based on *F*, with *F* set to zero for negative *F*^2^. The threshold expression of *F*^2^ > σ(*F*^2^) is used only for calculating *R*-factors(gt) *etc*. and is not relevant to the choice of reflections for refinement. *R*-factors based on *F*^2^ are statistically about twice as large as those based on *F*, and *R*- factors based on ALL data will be even larger.

### Fractional atomic coordinates and isotropic or equivalent isotropic displacement parameters (Å^2^)

**Table:**

	*x*	*y*	*z*	*U*_iso_*/*U*_eq_	
N1	0.92673 (14)	0.1214 (3)	0.79103 (16)	0.0488 (9)	
N2	0.87322 (13)	0.1356 (3)	0.75857 (14)	0.0466 (9)	
N3	0.61902 (14)	0.8466 (3)	0.73564 (16)	0.0515 (9)	
N4	0.56628 (15)	0.8716 (3)	0.70371 (17)	0.0541 (10)	
N5	0.45980 (15)	0.3732 (3)	0.81847 (16)	0.0521 (10)	
N6	0.49060 (14)	0.3495 (3)	0.87357 (15)	0.0481 (9)	
N7	0.95837 (15)	0.6256 (3)	0.83249 (16)	0.0529 (10)	
N8	0.99390 (14)	0.6439 (3)	0.88623 (15)	0.0503 (9)	
O1	0.63670 (12)	−0.0053 (3)	0.59691 (14)	0.0685 (10)	
O2	0.78167 (12)	0.2598 (3)	0.70625 (15)	0.0654 (9)	
H2	0.8137	0.2531	0.7252	0.098*	
O3	0.94118 (12)	0.3387 (3)	0.80046 (13)	0.0619 (9)	
O4	0.64814 (14)	0.3930 (3)	1.12381 (14)	0.0705 (10)	
O5	0.52907 (14)	0.2048 (3)	0.96401 (14)	0.0703 (10)	
H5A	0.5131	0.2210	0.9306	0.105*	
O6	0.45309 (12)	0.1576 (3)	0.79925 (13)	0.0605 (9)	
O7	0.86137 (14)	0.8658 (3)	0.89380 (16)	0.0833 (11)	
O8	0.70205 (13)	0.6939 (3)	0.77412 (16)	0.0712 (10)	
H8	0.6706	0.7141	0.7568	0.107*	
O9	0.54520 (12)	0.6564 (3)	0.70077 (13)	0.0615 (9)	
O10	1.16779 (14)	0.5194 (3)	1.12424 (13)	0.0752 (10)	
O11	1.04927 (15)	0.7739 (3)	0.97711 (14)	0.0723 (10)	
H11	1.0252	0.7631	0.9474	0.108*	
O12	0.95667 (12)	0.8398 (3)	0.80985 (13)	0.0582 (9)	
C1	0.68810 (16)	0.0131 (4)	0.63184 (19)	0.0489 (11)	
C2	0.70901 (16)	0.1315 (4)	0.65208 (18)	0.0502 (11)	
H2A	0.6879	0.2068	0.6424	0.060*	
C3	0.76194 (16)	0.1390 (4)	0.68715 (17)	0.0437 (10)	
C4	0.79378 (16)	0.0272 (4)	0.70278 (17)	0.0402 (10)	
C5	0.77056 (17)	−0.0912 (4)	0.68186 (19)	0.0545 (12)	
H5B	0.7909	−0.1674	0.6922	0.065*	
C6	0.71847 (18)	−0.0997 (4)	0.6463 (2)	0.0560 (12)	
H6	0.7039	−0.1802	0.6322	0.067*	
C7	0.60296 (19)	0.1050 (5)	0.5803 (2)	0.0708 (15)	
H7A	0.5965	0.1474	0.6140	0.106*	
H7B	0.5683	0.0783	0.5558	0.106*	
H7C	0.6211	0.1646	0.5597	0.106*	
C8	0.84853 (16)	0.0293 (4)	0.73875 (18)	0.0469 (11)	
H8A	0.8670	−0.0495	0.7482	0.056*	
C9	0.95849 (17)	0.2261 (4)	0.81013 (17)	0.0454 (11)	
C10	1.01542 (16)	0.1973 (4)	0.84336 (18)	0.0442 (10)	
C11	1.02782 (17)	0.0892 (4)	0.8785 (2)	0.0575 (13)	
H11A	1.0001	0.0298	0.8805	0.069*	
C12	1.08091 (18)	0.0682 (5)	0.9106 (2)	0.0622 (13)	
H12	1.0882	−0.0041	0.9348	0.075*	
C13	1.12305 (18)	0.1515 (6)	0.9075 (2)	0.0615 (13)	
C14	1.11118 (19)	0.2569 (5)	0.8716 (2)	0.0682 (15)	
H14	1.1394	0.3140	0.8685	0.082*	
C15	1.05785 (18)	0.2804 (5)	0.83961 (19)	0.0578 (12)	
H15	1.0507	0.3528	0.8155	0.069*	
C16	1.18103 (19)	0.1298 (6)	0.9441 (2)	0.0929 (19)	
H16A	1.2067	0.1826	0.9297	0.139*	
H16B	1.1908	0.0394	0.9425	0.139*	
H16C	1.1824	0.1537	0.9833	0.139*	
C17	0.70300 (17)	0.9248 (4)	0.79355 (18)	0.0465 (11)	
C18	0.72872 (18)	0.8012 (4)	0.80038 (19)	0.0517 (11)	
C19	0.78091 (19)	0.7856 (5)	0.8342 (2)	0.0606 (13)	
H19	0.7972	0.7033	0.8387	0.073*	
C20	0.80911 (18)	0.8915 (5)	0.8614 (2)	0.0577 (13)	
C21	0.78479 (19)	1.0141 (4)	0.8562 (2)	0.0613 (13)	
H21	0.8037	1.0856	0.8753	0.074*	
C22	0.73255 (17)	1.0281 (5)	0.8224 (2)	0.0571 (12)	
H22	0.7163	1.1105	0.8187	0.069*	
C23	0.8935 (2)	0.9669 (5)	0.9235 (2)	0.0862 (18)	
H23A	0.9003	1.0306	0.8964	0.129*	
H23B	0.9280	0.9325	0.9451	0.129*	
H23C	0.8742	1.0074	0.9493	0.129*	
C24	0.64784 (17)	0.9434 (4)	0.75882 (19)	0.0512 (12)	
H24	0.6331	1.0273	0.7533	0.061*	
C25	0.53174 (18)	0.7693 (4)	0.68643 (19)	0.0489 (11)	
C26	0.47687 (18)	0.8038 (4)	0.65010 (18)	0.0480 (11)	
C27	0.43139 (19)	0.7321 (5)	0.6551 (2)	0.0593 (13)	
H27	0.4355	0.6644	0.6818	0.071*	
C28	0.37977 (19)	0.7599 (5)	0.6207 (2)	0.0659 (14)	
H28	0.3494	0.7110	0.6247	0.079*	
C29	0.37244 (19)	0.8584 (5)	0.5806 (2)	0.0605 (13)	
C30	0.4176 (2)	0.9289 (5)	0.5751 (2)	0.0643 (14)	
H30	0.4136	0.9947	0.5475	0.077*	
C31	0.4689 (2)	0.9031 (5)	0.6101 (2)	0.0618 (13)	
H31	0.4989	0.9540	0.6066	0.074*	
C32	0.3155 (2)	0.8866 (6)	0.5430 (2)	0.0922 (19)	
H32A	0.3082	0.8288	0.5104	0.138*	
H32B	0.3140	0.9755	0.5299	0.138*	
H32C	0.2882	0.8735	0.5650	0.138*	
C33	0.54701 (16)	0.4341 (4)	0.95954 (18)	0.0438 (11)	
C34	0.55453 (17)	0.3140 (4)	0.9892 (2)	0.0498 (11)	
C35	0.58775 (17)	0.3055 (4)	1.0436 (2)	0.0573 (12)	
H35	0.5920	0.2254	1.0625	0.069*	
C36	0.61482 (18)	0.4123 (4)	1.07044 (19)	0.0499 (11)	
C37	0.60840 (18)	0.5329 (4)	1.04267 (19)	0.0569 (12)	
H37	0.6269	0.6063	1.0606	0.068*	
C38	0.57441 (19)	0.5417 (4)	0.9885 (2)	0.0573 (12)	
H38	0.5694	0.6228	0.9705	0.069*	
C39	0.51311 (17)	0.4483 (4)	0.90242 (18)	0.0484 (11)	
H39	0.5074	0.5311	0.8860	0.058*	
C40	0.44205 (16)	0.2700 (4)	0.78324 (19)	0.0451 (11)	
C41	0.40733 (16)	0.3021 (4)	0.72540 (19)	0.0458 (11)	
C42	0.40758 (18)	0.2181 (5)	0.6802 (2)	0.0574 (12)	
H42	0.4310	0.1459	0.6860	0.069*	
C43	0.3734 (2)	0.2406 (5)	0.6267 (2)	0.0678 (14)	
H43	0.3739	0.1826	0.5968	0.081*	
C44	0.3386 (2)	0.3463 (6)	0.6163 (2)	0.0623 (13)	
C45	0.3381 (2)	0.4285 (5)	0.6614 (2)	0.0661 (14)	
H45	0.3135	0.4983	0.6559	0.079*	
C46	0.37298 (18)	0.4103 (4)	0.7145 (2)	0.0567 (12)	
H46	0.3736	0.4713	0.7436	0.068*	
C47	0.3009 (2)	0.3688 (7)	0.5577 (2)	0.099 (2)	
H47A	0.3214	0.3578	0.5286	0.148*	
H47B	0.2863	0.4559	0.5557	0.148*	
H47C	0.2711	0.3070	0.5514	0.148*	
C48	0.6785 (2)	0.4992 (5)	1.1522 (2)	0.0756 (15)	
H48A	0.7000	0.5371	1.1279	0.113*	
H48B	0.7027	0.4696	1.1874	0.113*	
H48C	0.6535	0.5635	1.1609	0.113*	
C49	1.05240 (17)	0.5418 (4)	0.96794 (18)	0.0460 (11)	
C50	1.06956 (18)	0.6555 (4)	0.99853 (18)	0.0486 (11)	
C51	1.10784 (19)	0.6509 (4)	1.05129 (18)	0.0554 (12)	
H51	1.1189	0.7275	1.0718	0.066*	
C52	1.12932 (19)	0.5329 (4)	1.07304 (19)	0.0546 (12)	
C53	1.1133 (2)	0.4190 (4)	1.0437 (2)	0.0634 (14)	
H53	1.1283	0.3394	1.0585	0.076*	
C54	1.07462 (19)	0.4243 (4)	0.99216 (19)	0.0600 (13)	
H54	1.0629	0.3468	0.9728	0.072*	
C55	1.1878 (2)	0.6325 (5)	1.1558 (2)	0.0731 (15)	
H55A	1.2062	0.6869	1.1333	0.110*	
H55B	1.2134	0.6083	1.1910	0.110*	
H55C	1.1574	0.6796	1.1646	0.110*	
C56	1.01301 (17)	0.5393 (4)	0.91320 (18)	0.0492 (11)	
H56	1.0010	0.4589	0.8967	0.059*	
C57	0.94209 (17)	0.7264 (4)	0.79666 (19)	0.0481 (11)	
C58	0.90607 (16)	0.6916 (4)	0.74035 (19)	0.0455 (11)	
C59	0.86713 (17)	0.5928 (4)	0.73383 (19)	0.0533 (12)	
H59	0.8633	0.5457	0.7660	0.064*	
C60	0.83402 (18)	0.5632 (4)	0.6807 (2)	0.0582 (13)	
H60	0.8074	0.4980	0.6775	0.070*	
C61	0.83967 (19)	0.6291 (5)	0.6319 (2)	0.0607 (13)	
C62	0.8788 (2)	0.7253 (5)	0.6380 (2)	0.0679 (14)	
H62	0.8831	0.7709	0.6056	0.081*	
C63	0.91227 (17)	0.7563 (5)	0.6915 (2)	0.0561 (12)	
H63	0.9390	0.8210	0.6945	0.067*	
C64	0.8032 (2)	0.5978 (6)	0.5733 (2)	0.0919 (19)	
H64A	0.7713	0.6537	0.5660	0.138*	
H64B	0.7916	0.5082	0.5725	0.138*	
H64C	0.8235	0.6117	0.5443	0.138*	
H4	0.5578 (19)	0.9566 (15)	0.699 (2)	0.080*	
H5	0.4583 (19)	0.4573 (16)	0.8081 (19)	0.080*	
H1	0.9400 (18)	0.0397 (19)	0.796 (2)	0.080*	
H7	0.9532 (19)	0.5417 (17)	0.8220 (19)	0.080*	

### Atomic displacement parameters (Å^2^)

**Table:**

	*U*^11^	*U*^22^	*U*^33^	*U*^12^	*U*^13^	*U*^23^
N1	0.043 (2)	0.034 (2)	0.057 (2)	−0.0033 (17)	−0.0111 (18)	−0.0006 (18)
N2	0.042 (2)	0.042 (2)	0.050 (2)	0.0001 (17)	−0.0025 (17)	0.0000 (18)
N3	0.050 (2)	0.037 (2)	0.063 (2)	−0.0019 (18)	0.0037 (19)	0.0036 (19)
N4	0.050 (2)	0.039 (2)	0.066 (3)	−0.0056 (19)	−0.002 (2)	0.001 (2)
N5	0.060 (2)	0.038 (2)	0.052 (2)	0.0041 (19)	0.001 (2)	0.0029 (19)
N6	0.047 (2)	0.043 (2)	0.048 (2)	0.0000 (17)	−0.0017 (18)	−0.0030 (18)
N7	0.060 (2)	0.038 (2)	0.051 (2)	−0.0037 (19)	−0.005 (2)	0.0065 (19)
N8	0.053 (2)	0.043 (2)	0.049 (2)	0.0020 (18)	−0.0011 (19)	0.0088 (18)
O1	0.0408 (18)	0.057 (2)	0.092 (3)	−0.0017 (16)	−0.0155 (17)	−0.0038 (18)
O2	0.059 (2)	0.0347 (17)	0.088 (3)	0.0022 (15)	−0.0122 (18)	−0.0086 (17)
O3	0.066 (2)	0.0397 (19)	0.068 (2)	0.0004 (16)	−0.0092 (17)	−0.0013 (16)
O4	0.081 (2)	0.056 (2)	0.059 (2)	−0.0042 (18)	−0.0145 (19)	0.0089 (17)
O5	0.076 (2)	0.0407 (19)	0.080 (3)	−0.0102 (17)	−0.011 (2)	0.0017 (17)
O6	0.070 (2)	0.0338 (18)	0.068 (2)	−0.0023 (15)	−0.0038 (17)	0.0004 (16)
O7	0.064 (2)	0.064 (2)	0.104 (3)	−0.0027 (19)	−0.016 (2)	0.018 (2)
O8	0.071 (2)	0.0381 (18)	0.094 (3)	−0.0002 (16)	−0.002 (2)	−0.0062 (18)
O9	0.070 (2)	0.0371 (19)	0.070 (2)	−0.0054 (16)	0.0027 (17)	−0.0003 (16)
O10	0.099 (3)	0.058 (2)	0.050 (2)	0.0105 (19)	−0.0189 (19)	0.0015 (17)
O11	0.092 (3)	0.0378 (19)	0.071 (2)	0.0067 (17)	−0.0133 (19)	0.0005 (17)
O12	0.0564 (19)	0.0374 (19)	0.069 (2)	−0.0004 (15)	−0.0086 (16)	0.0022 (16)
C1	0.033 (2)	0.050 (3)	0.057 (3)	−0.002 (2)	−0.001 (2)	−0.002 (2)
C2	0.044 (3)	0.038 (3)	0.061 (3)	0.008 (2)	−0.003 (2)	−0.002 (2)
C3	0.048 (3)	0.032 (2)	0.047 (3)	−0.004 (2)	0.005 (2)	−0.007 (2)
C4	0.038 (2)	0.034 (2)	0.045 (3)	−0.0002 (19)	0.0034 (19)	−0.006 (2)
C5	0.051 (3)	0.037 (3)	0.072 (3)	0.003 (2)	0.006 (2)	−0.005 (2)
C6	0.053 (3)	0.035 (3)	0.070 (3)	−0.005 (2)	−0.005 (2)	−0.010 (2)
C7	0.051 (3)	0.073 (4)	0.080 (4)	0.012 (3)	−0.001 (3)	0.000 (3)
C8	0.048 (3)	0.036 (2)	0.053 (3)	0.001 (2)	0.005 (2)	−0.001 (2)
C9	0.048 (3)	0.040 (3)	0.042 (3)	−0.001 (2)	−0.003 (2)	0.000 (2)
C10	0.042 (2)	0.040 (2)	0.047 (3)	−0.008 (2)	0.003 (2)	−0.005 (2)
C11	0.043 (3)	0.049 (3)	0.070 (3)	−0.010 (2)	−0.007 (2)	0.004 (3)
C12	0.055 (3)	0.059 (3)	0.061 (3)	−0.003 (2)	−0.011 (2)	0.004 (3)
C13	0.044 (3)	0.085 (4)	0.051 (3)	−0.002 (3)	0.003 (2)	−0.011 (3)
C14	0.050 (3)	0.089 (4)	0.066 (4)	−0.022 (3)	0.017 (3)	−0.003 (3)
C15	0.057 (3)	0.063 (3)	0.053 (3)	−0.012 (3)	0.013 (2)	0.003 (2)
C16	0.053 (3)	0.137 (6)	0.080 (4)	0.000 (3)	−0.001 (3)	0.001 (4)
C17	0.051 (3)	0.031 (2)	0.055 (3)	−0.003 (2)	0.008 (2)	−0.001 (2)
C18	0.058 (3)	0.037 (3)	0.058 (3)	−0.004 (2)	0.010 (2)	0.002 (2)
C19	0.063 (3)	0.044 (3)	0.070 (3)	0.009 (2)	0.006 (3)	0.011 (3)
C20	0.047 (3)	0.056 (3)	0.063 (3)	0.001 (2)	−0.002 (2)	0.017 (3)
C21	0.055 (3)	0.042 (3)	0.082 (4)	−0.013 (2)	0.007 (3)	−0.005 (3)
C22	0.047 (3)	0.044 (3)	0.077 (3)	0.003 (2)	0.007 (3)	−0.004 (3)
C23	0.062 (3)	0.086 (4)	0.097 (4)	−0.018 (3)	−0.008 (3)	0.010 (4)
C24	0.051 (3)	0.037 (3)	0.063 (3)	0.005 (2)	0.009 (2)	0.006 (2)
C25	0.057 (3)	0.039 (3)	0.051 (3)	−0.008 (2)	0.013 (2)	−0.010 (2)
C26	0.055 (3)	0.037 (2)	0.050 (3)	−0.002 (2)	0.010 (2)	−0.002 (2)
C27	0.062 (3)	0.060 (3)	0.055 (3)	−0.009 (3)	0.013 (3)	−0.003 (2)
C28	0.052 (3)	0.083 (4)	0.064 (3)	−0.011 (3)	0.016 (3)	−0.006 (3)
C29	0.052 (3)	0.073 (4)	0.052 (3)	0.001 (3)	0.002 (2)	−0.009 (3)
C30	0.067 (3)	0.058 (3)	0.060 (3)	0.002 (3)	−0.001 (3)	0.003 (3)
C31	0.059 (3)	0.053 (3)	0.067 (3)	−0.014 (2)	0.004 (3)	0.002 (3)
C32	0.061 (3)	0.120 (5)	0.082 (4)	0.012 (3)	−0.011 (3)	−0.006 (4)
C33	0.046 (2)	0.036 (3)	0.044 (3)	0.0043 (19)	0.000 (2)	−0.001 (2)
C34	0.046 (3)	0.033 (2)	0.065 (3)	−0.004 (2)	0.003 (2)	0.000 (2)
C35	0.055 (3)	0.041 (3)	0.066 (3)	0.005 (2)	−0.004 (3)	0.012 (2)
C36	0.054 (3)	0.041 (3)	0.050 (3)	0.004 (2)	0.000 (2)	0.003 (2)
C37	0.065 (3)	0.041 (3)	0.056 (3)	−0.004 (2)	−0.004 (2)	−0.005 (2)
C38	0.075 (3)	0.033 (3)	0.059 (3)	0.004 (2)	0.006 (3)	0.009 (2)
C39	0.056 (3)	0.035 (3)	0.053 (3)	0.007 (2)	0.011 (2)	0.001 (2)
C40	0.041 (2)	0.032 (3)	0.059 (3)	−0.004 (2)	0.007 (2)	−0.002 (2)
C41	0.040 (2)	0.045 (3)	0.050 (3)	−0.002 (2)	0.005 (2)	0.000 (2)
C42	0.054 (3)	0.057 (3)	0.060 (3)	−0.004 (2)	0.010 (3)	−0.009 (3)
C43	0.076 (4)	0.072 (4)	0.056 (3)	−0.013 (3)	0.015 (3)	−0.017 (3)
C44	0.059 (3)	0.078 (4)	0.045 (3)	−0.017 (3)	0.004 (2)	0.005 (3)
C45	0.063 (3)	0.067 (4)	0.062 (3)	0.007 (3)	0.001 (3)	0.010 (3)
C46	0.059 (3)	0.048 (3)	0.055 (3)	0.005 (2)	−0.004 (2)	−0.004 (2)
C47	0.091 (4)	0.129 (6)	0.068 (4)	−0.002 (4)	0.002 (3)	0.008 (4)
C48	0.083 (4)	0.071 (4)	0.062 (3)	0.000 (3)	−0.003 (3)	−0.003 (3)
C49	0.054 (3)	0.035 (2)	0.045 (3)	0.000 (2)	0.005 (2)	0.004 (2)
C50	0.062 (3)	0.034 (3)	0.045 (3)	0.002 (2)	0.004 (2)	0.002 (2)
C51	0.076 (3)	0.036 (3)	0.047 (3)	0.004 (2)	0.001 (3)	−0.004 (2)
C52	0.072 (3)	0.043 (3)	0.042 (3)	0.006 (2)	0.001 (2)	0.001 (2)
C53	0.088 (4)	0.040 (3)	0.052 (3)	0.010 (3)	−0.005 (3)	0.010 (2)
C54	0.081 (3)	0.040 (3)	0.053 (3)	0.001 (2)	0.003 (3)	−0.002 (2)
C55	0.080 (4)	0.075 (4)	0.052 (3)	0.002 (3)	−0.011 (3)	−0.017 (3)
C56	0.057 (3)	0.039 (3)	0.048 (3)	−0.007 (2)	0.004 (2)	−0.001 (2)
C57	0.043 (2)	0.040 (3)	0.057 (3)	0.001 (2)	0.004 (2)	0.011 (2)
C58	0.039 (2)	0.039 (2)	0.055 (3)	0.004 (2)	0.004 (2)	0.006 (2)
C59	0.053 (3)	0.047 (3)	0.053 (3)	−0.001 (2)	−0.001 (2)	0.011 (2)
C60	0.053 (3)	0.040 (3)	0.072 (4)	−0.004 (2)	−0.005 (3)	−0.002 (3)
C61	0.055 (3)	0.070 (3)	0.051 (3)	0.012 (3)	0.001 (2)	−0.004 (3)
C62	0.059 (3)	0.089 (4)	0.052 (3)	0.004 (3)	0.005 (3)	0.019 (3)
C63	0.045 (3)	0.061 (3)	0.063 (3)	0.000 (2)	0.015 (2)	0.010 (3)
C64	0.082 (4)	0.124 (5)	0.057 (4)	−0.001 (4)	−0.009 (3)	−0.003 (3)

### Geometric parameters (Å, º)

**Table:**

N1—C9	1.345 (5)	C23—H23B	0.9600
N1—N2	1.380 (4)	C23—H23C	0.9600
N1—H1	0.897 (10)	C24—H24	0.9300
N2—C8	1.283 (5)	C25—C26	1.477 (6)
N3—C24	1.271 (5)	C26—C31	1.376 (6)
N3—N4	1.377 (5)	C26—C27	1.377 (6)
N4—C25	1.355 (5)	C27—C28	1.381 (6)
N4—H4	0.897 (10)	C27—H27	0.9300
N5—C40	1.357 (5)	C28—C29	1.371 (6)
N5—N6	1.378 (5)	C28—H28	0.9300
N5—H5	0.894 (10)	C29—C30	1.368 (6)
N6—C39	1.275 (5)	C29—C32	1.514 (6)
N7—C57	1.340 (5)	C30—C31	1.375 (6)
N7—N8	1.387 (5)	C30—H30	0.9300
N7—H7	0.896 (10)	C31—H31	0.9300
N8—C56	1.282 (5)	C32—H32A	0.9600
O1—C1	1.364 (5)	C32—H32B	0.9600
O1—C7	1.407 (5)	C32—H32C	0.9600
O2—C3	1.368 (4)	C33—C38	1.390 (6)
O2—H2	0.8200	C33—C34	1.409 (5)
O3—C9	1.233 (5)	C33—C39	1.431 (5)
O4—C36	1.360 (5)	C34—C35	1.365 (6)
O4—C48	1.403 (5)	C35—C36	1.363 (6)
O5—C34	1.354 (5)	C35—H35	0.9300
O5—H5A	0.8200	C36—C37	1.392 (6)
O6—C40	1.222 (5)	C37—C38	1.369 (6)
O7—C20	1.372 (5)	C37—H37	0.9300
O7—C23	1.394 (6)	C38—H38	0.9300
O8—C18	1.357 (5)	C39—H39	0.9300
O8—H8	0.8200	C40—C41	1.482 (6)
O9—C25	1.229 (5)	C41—C42	1.380 (6)
O10—C52	1.369 (5)	C41—C46	1.386 (6)
O10—C55	1.406 (5)	C42—C43	1.377 (6)
O11—C50	1.365 (5)	C42—H42	0.9300
O11—H11	0.8200	C43—C44	1.370 (7)
O12—C57	1.235 (5)	C43—H43	0.9300
C1—C2	1.362 (6)	C44—C45	1.368 (7)
C1—C6	1.378 (6)	C44—C47	1.507 (6)
C2—C3	1.385 (5)	C45—C46	1.368 (6)
C2—H2A	0.9300	C45—H45	0.9300
C3—C4	1.391 (5)	C46—H46	0.9300
C4—C5	1.384 (5)	C47—H47A	0.9600
C4—C8	1.428 (5)	C47—H47B	0.9600
C5—C6	1.374 (5)	C47—H47C	0.9600
C5—H5B	0.9300	C48—H48A	0.9600
C6—H6	0.9300	C48—H48B	0.9600
C7—H7A	0.9600	C48—H48C	0.9600
C7—H7B	0.9600	C49—C50	1.386 (5)
C7—H7C	0.9600	C49—C54	1.391 (6)
C8—H8A	0.9300	C49—C56	1.436 (5)
C9—C10	1.480 (5)	C50—C51	1.388 (5)
C10—C15	1.374 (5)	C51—C52	1.372 (6)
C10—C11	1.378 (6)	C51—H51	0.9300
C11—C12	1.378 (6)	C52—C53	1.370 (6)
C11—H11A	0.9300	C53—C54	1.371 (6)
C12—C13	1.366 (6)	C53—H53	0.9300
C12—H12	0.9300	C54—H54	0.9300
C13—C14	1.367 (7)	C55—H55A	0.9600
C13—C16	1.516 (6)	C55—H55B	0.9600
C14—C15	1.386 (6)	C55—H55C	0.9600
C14—H14	0.9300	C56—H56	0.9300
C15—H15	0.9300	C57—C58	1.472 (6)
C16—H16A	0.9600	C58—C63	1.380 (6)
C16—H16B	0.9600	C58—C59	1.383 (6)
C16—H16C	0.9600	C59—C60	1.373 (6)
C17—C22	1.376 (6)	C59—H59	0.9300
C17—C18	1.409 (6)	C60—C61	1.381 (6)
C17—C24	1.438 (6)	C60—H60	0.9300
C18—C19	1.366 (6)	C61—C62	1.369 (7)
C19—C20	1.369 (6)	C61—C64	1.510 (6)
C19—H19	0.9300	C62—C63	1.385 (6)
C20—C21	1.386 (6)	C62—H62	0.9300
C21—C22	1.365 (6)	C63—H63	0.9300
C21—H21	0.9300	C64—H64A	0.9600
C22—H22	0.9300	C64—H64B	0.9600
C23—H23A	0.9600	C64—H64C	0.9600
			
C9—N1—N2	121.2 (3)	C29—C30—C31	120.4 (5)
C9—N1—H1	122 (3)	C29—C30—H30	119.8
N2—N1—H1	117 (3)	C31—C30—H30	119.8
C8—N2—N1	115.6 (3)	C30—C31—C26	121.6 (5)
C24—N3—N4	117.5 (4)	C30—C31—H31	119.2
C25—N4—N3	118.5 (4)	C26—C31—H31	119.2
C25—N4—H4	127 (3)	C29—C32—H32A	109.5
N3—N4—H4	115 (3)	C29—C32—H32B	109.5
C40—N5—N6	118.7 (4)	H32A—C32—H32B	109.5
C40—N5—H5	126 (3)	C29—C32—H32C	109.5
N6—N5—H5	114 (3)	H32A—C32—H32C	109.5
C39—N6—N5	116.7 (4)	H32B—C32—H32C	109.5
C57—N7—N8	121.1 (4)	C38—C33—C34	116.7 (4)
C57—N7—H7	124 (3)	C38—C33—C39	120.2 (4)
N8—N7—H7	114 (3)	C34—C33—C39	123.1 (4)
C56—N8—N7	115.5 (4)	O5—C34—C35	118.9 (4)
C1—O1—C7	118.2 (4)	O5—C34—C33	120.3 (4)
C3—O2—H2	109.5	C35—C34—C33	120.8 (4)
C36—O4—C48	118.6 (4)	C36—C35—C34	121.1 (4)
C34—O5—H5A	109.5	C36—C35—H35	119.5
C20—O7—C23	119.8 (4)	C34—C35—H35	119.5
C18—O8—H8	109.5	O4—C36—C35	116.6 (4)
C52—O10—C55	118.6 (4)	O4—C36—C37	123.4 (4)
C50—O11—H11	109.5	C35—C36—C37	119.9 (4)
C2—C1—O1	124.3 (4)	C38—C37—C36	118.9 (4)
C2—C1—C6	121.1 (4)	C38—C37—H37	120.6
O1—C1—C6	114.6 (4)	C36—C37—H37	120.6
C1—C2—C3	119.5 (4)	C37—C38—C33	122.6 (4)
C1—C2—H2A	120.3	C37—C38—H38	118.7
C3—C2—H2A	120.3	C33—C38—H38	118.7
O2—C3—C2	117.8 (4)	N6—C39—C33	121.2 (4)
O2—C3—C4	121.1 (3)	N6—C39—H39	119.4
C2—C3—C4	121.1 (4)	C33—C39—H39	119.4
C5—C4—C3	117.4 (4)	O6—C40—N5	121.5 (4)
C5—C4—C8	119.2 (4)	O6—C40—C41	122.6 (4)
C3—C4—C8	123.4 (4)	N5—C40—C41	115.9 (4)
C6—C5—C4	122.0 (4)	C42—C41—C46	117.9 (4)
C6—C5—H5B	119.0	C42—C41—C40	118.7 (4)
C4—C5—H5B	119.0	C46—C41—C40	123.4 (4)
C5—C6—C1	118.9 (4)	C43—C42—C41	120.3 (5)
C5—C6—H6	120.6	C43—C42—H42	119.8
C1—C6—H6	120.6	C41—C42—H42	119.8
O1—C7—H7A	109.5	C44—C43—C42	121.6 (5)
O1—C7—H7B	109.5	C44—C43—H43	119.2
H7A—C7—H7B	109.5	C42—C43—H43	119.2
O1—C7—H7C	109.5	C45—C44—C43	117.8 (5)
H7A—C7—H7C	109.5	C45—C44—C47	121.1 (5)
H7B—C7—H7C	109.5	C43—C44—C47	121.0 (5)
N2—C8—C4	122.7 (4)	C44—C45—C46	121.5 (5)
N2—C8—H8A	118.7	C44—C45—H45	119.2
C4—C8—H8A	118.7	C46—C45—H45	119.2
O3—C9—N1	122.0 (4)	C45—C46—C41	120.7 (5)
O3—C9—C10	122.3 (4)	C45—C46—H46	119.7
N1—C9—C10	115.7 (4)	C41—C46—H46	119.7
C15—C10—C11	118.1 (4)	C44—C47—H47A	109.5
C15—C10—C9	119.6 (4)	C44—C47—H47B	109.5
C11—C10—C9	122.3 (4)	H47A—C47—H47B	109.5
C10—C11—C12	120.7 (4)	C44—C47—H47C	109.5
C10—C11—H11A	119.6	H47A—C47—H47C	109.5
C12—C11—H11A	119.6	H47B—C47—H47C	109.5
C13—C12—C11	121.3 (5)	O4—C48—H48A	109.5
C13—C12—H12	119.4	O4—C48—H48B	109.5
C11—C12—H12	119.4	H48A—C48—H48B	109.5
C12—C13—C14	118.1 (4)	O4—C48—H48C	109.5
C12—C13—C16	121.0 (5)	H48A—C48—H48C	109.5
C14—C13—C16	120.8 (5)	H48B—C48—H48C	109.5
C13—C14—C15	121.3 (5)	C50—C49—C54	117.7 (4)
C13—C14—H14	119.4	C50—C49—C56	123.6 (4)
C15—C14—H14	119.4	C54—C49—C56	118.8 (4)
C10—C15—C14	120.5 (5)	O11—C50—C49	120.5 (4)
C10—C15—H15	119.8	O11—C50—C51	118.9 (4)
C14—C15—H15	119.8	C49—C50—C51	120.6 (4)
C13—C16—H16A	109.5	C52—C51—C50	119.7 (4)
C13—C16—H16B	109.5	C52—C51—H51	120.2
H16A—C16—H16B	109.5	C50—C51—H51	120.2
C13—C16—H16C	109.5	O10—C52—C53	115.3 (4)
H16A—C16—H16C	109.5	O10—C52—C51	123.6 (4)
H16B—C16—H16C	109.5	C53—C52—C51	121.0 (4)
C22—C17—C18	117.2 (4)	C52—C53—C54	118.8 (4)
C22—C17—C24	120.7 (4)	C52—C53—H53	120.6
C18—C17—C24	122.1 (4)	C54—C53—H53	120.6
O8—C18—C19	118.1 (4)	C53—C54—C49	122.2 (4)
O8—C18—C17	120.9 (4)	C53—C54—H54	118.9
C19—C18—C17	121.0 (4)	C49—C54—H54	118.9
C18—C19—C20	119.7 (4)	O10—C55—H55A	109.5
C18—C19—H19	120.1	O10—C55—H55B	109.5
C20—C19—H19	120.1	H55A—C55—H55B	109.5
C19—C20—O7	115.2 (4)	O10—C55—H55C	109.5
C19—C20—C21	120.7 (4)	H55A—C55—H55C	109.5
O7—C20—C21	124.0 (5)	H55B—C55—H55C	109.5
C22—C21—C20	118.8 (4)	N8—C56—C49	122.3 (4)
C22—C21—H21	120.6	N8—C56—H56	118.9
C20—C21—H21	120.6	C49—C56—H56	118.9
C21—C22—C17	122.5 (4)	O12—C57—N7	122.3 (4)
C21—C22—H22	118.8	O12—C57—C58	122.6 (4)
C17—C22—H22	118.8	N7—C57—C58	115.0 (4)
O7—C23—H23A	109.5	C63—C58—C59	118.0 (4)
O7—C23—H23B	109.5	C63—C58—C57	119.3 (4)
H23A—C23—H23B	109.5	C59—C58—C57	122.6 (4)
O7—C23—H23C	109.5	C60—C59—C58	121.1 (4)
H23A—C23—H23C	109.5	C60—C59—H59	119.4
H23B—C23—H23C	109.5	C58—C59—H59	119.4
N3—C24—C17	120.7 (4)	C59—C60—C61	120.8 (4)
N3—C24—H24	119.7	C59—C60—H60	119.6
C17—C24—H24	119.7	C61—C60—H60	119.6
O9—C25—N4	122.0 (4)	C62—C61—C60	118.3 (4)
O9—C25—C26	122.8 (4)	C62—C61—C64	120.5 (5)
N4—C25—C26	115.2 (4)	C60—C61—C64	121.2 (5)
C31—C26—C27	117.8 (4)	C61—C62—C63	121.3 (5)
C31—C26—C25	123.1 (4)	C61—C62—H62	119.3
C27—C26—C25	119.1 (4)	C63—C62—H62	119.3
C26—C27—C28	120.4 (5)	C58—C63—C62	120.4 (4)
C26—C27—H27	119.8	C58—C63—H63	119.8
C28—C27—H27	119.8	C62—C63—H63	119.8
C29—C28—C27	121.2 (5)	C61—C64—H64A	109.5
C29—C28—H28	119.4	C61—C64—H64B	109.5
C27—C28—H28	119.4	H64A—C64—H64B	109.5
C30—C29—C28	118.6 (4)	C61—C64—H64C	109.5
C30—C29—C32	121.1 (5)	H64A—C64—H64C	109.5
C28—C29—C32	120.3 (5)	H64B—C64—H64C	109.5

### Hydrogen-bond geometry (Å, º)

**Table:**

*D*—H···*A*	*D*—H	H···*A*	*D*···*A*	*D*—H···*A*
O2—H2···N2	0.82	1.93	2.649 (4)	146
O5—H5*A*···N6	0.82	1.88	2.606 (5)	147
O8—H8···N3	0.82	1.85	2.580 (4)	147
O11—H11···N8	0.82	1.92	2.638 (5)	146
N4—H4···O6^i^	0.90 (1)	2.08 (1)	2.965 (5)	171 (4)
N5—H5···O9^ii^	0.89 (1)	2.05 (1)	2.932 (5)	170 (4)
N1—H1···O12^iii^	0.90 (1)	2.10 (1)	2.984 (4)	169 (4)
N7—H7···O3	0.90 (1)	2.14 (1)	3.039 (5)	178 (4)
C6—H6···O4^iv^	0.93	2.56	3.452 (5)	160 (4)
C24—H24···O6^i^	0.93	2.58	3.374 (5)	143 (4)
C35—H35···O1^v^	0.93	2.56	3.436 (5)	157 (4)
C39—H39···O9^ii^	0.93	2.52	3.319 (5)	144 (4)
C19—H19···O10^vi^	0.93	2.52	3.429 (5)	164 (4)
C53—H53···O7^vi^	0.93	2.37	3.266 (5)	161 (4)

Symmetry codes: (i) −*x*+1, *y*+1, −*z*+3/2; (ii) −*x*+1, *y*, −*z*+3/2; (iii) *x*, *y*−1, *z*; (iv) *x*, −*y*, *z*−1/2; (v) *x*, −*y*, *z*+1/2; (vi) −*x*+2, −*y*+1, −*z*+2.
